# Cryptococcal Meningitis Confounded by Concurrent Cytomegalovirus Viraemia in a Renal Transplant Recipient: A Diagnostic Challenge

**DOI:** 10.7759/cureus.108538

**Published:** 2026-05-09

**Authors:** Mehadi Hasan, Sanjida Sharmin, Yosef Mustafa, Lauren Kandakumar, Abdullah Al Hossain Nidul, Shahneela Shakoor

**Affiliations:** 1 Internal Medicine, Evercare Hospital Dhaka, Dhaka, BGD; 2 Obstetrics and Gynaecology, Dhaka Medical College Hospital, Dhaka, BGD; 3 Medical Education, National Health Service, London, GBR; 4 General Medicine, Southend University Hospital, Southend-on-Sea, GBR; 5 Critical Care, Dhaka National Medical Institute Hospital, Dhaka, BGD; 6 General Medicine, Mid and South Essex NHS Foundation Trust, Southend-on-Sea, GBR

**Keywords:** cmv, cryptococcal infection, immunosuppressants, opportunistic infection, renal transplant

## Abstract

Cryptococcal meningitis is a well-recognized opportunistic infection in immunocompromised patients, especially in transplant recipients receiving immunosuppressive therapy. Concurrent cytomegalovirus (CMV) viraemia is uncommon and can make the clinical picture misleading. We report a renal transplant recipient who presented with fever, headache, and generalized malaise. He was initially diagnosed with CMV viraemia and started on valganciclovir. However, his condition later worsened, and he developed seizures. Further investigation revealed cryptococcal meningitis, confirmed by cerebrospinal fluid culture. This case highlights the need for a high index of suspicion in immunosuppressed patients. It also emphasizes the importance of considering dual opportunistic infections when symptoms persist or worsen despite appropriate treatment.

## Introduction

Cryptococcal meningitis is a life-threatening fungal infection, particularly in patients with impaired immunity, such as organ transplant recipients and those with acquired immunodeficiency syndrome (AIDS), diabetes, and sickle cell disease [1]. Renal transplant recipients remain particularly susceptible to opportunistic infections due to ongoing immunosuppressive therapy [2]. The diagnosis may be challenging when symptoms are non-specific or when more than one infectious process is present at the same time. Cryptococcal meningitis is a serious but potentially under-recognized infection in this group, especially when concurrent findings such as cytomegalovirus (CMV) viraemia offer an alternative explanation for the presentation [2]. We present a case that illustrates the diagnostic difficulty created by such overlapping opportunistic infections.

## Case presentation

A 69-year-old man presented with complaints of fever, diarrhoea, persistent headache, weakness, and vertigo for two weeks. His fever was high-grade and intermittent and subsided with antipyretics. He described his headache as insidious in onset, dull-aching, persistent, and not fully relieved by over-the-counter analgesics. He denied any photophobia, phonophobia, neck stiffness, or rash associated with headaches. He was a known case of diabetes and hypertension and had undergone renal transplantation two years earlier. On examination, his vitals were within normal range apart from high-grade temperature. His full neurological system, including cranial nerve examinations, was normal with no signs of meningism. Fundoscopy revealed diabetic changes in both eyes. He was taking several immunosuppressants, including tacrolimus 2 mg twice daily, mycophenolate mofetil 500 mg twice daily, and prednisolone 5 mg once daily. He was compliant with his regular medication. A series of lab investigations was performed.

His initial lab test revealed mild anaemia (Table 1) and normal renal function with hyponatraemia (Table 2).

**Table 1:** Initial full blood count demonstrating haemoglobin and WBC count below the reference range with platelet count within normal limits RBC: red blood cell; WBC: white blood cell

Test name	Result	Reference range
Haemoglobin	10.6 gm/dl	13.5-17.5 gm/dl
RBC count	3.98 million/L	4.5-5.5 million/L
WBC count	3.94 K/dl	4-11 K/dl
Platelet count	155 K/dl	150-400 K/dl

**Table 2:** Complete renal profile demonstrating normal renal function with hyponatraemia

Test name	Result	Reference range
Urea	27 mg/dl	15-45 mg/dl
Creatinine	1.3 mg/dl	0.6-1.3 mg/dl
Sodium (Na+)	125 mmol/L	135-145 mmol/L
Potassium (K+)	3.6 mmol/L	3.5-5 mmol/L
Chloride (Cl-)	91 mmol/L	98-108 mmol/L
Bicarbonate (HCO3-)	27 mmol/L	24-32 mmol/L
Uric acid	3.3 mg/dl	3-7.2 mg/dl
Serum albumin	3.6 gm/dl	3.5-5 gm/dl
Calcium	9 mg/dl	8.5-10.5 mg/dl
Phosphate	2.5 mg/dl	2.5-4.5 mg/dl

Serological tests for hepatitis B and C, including human immunodeficiency virus (HIV), were negative. His blood, urine, and stool cultures didn't reveal any growth. Chest X-ray and abdominal ultrasound (USG) were also normal. His blood sample was sent for CMV reverse transcription polymerase chain reaction (RT-PCR) (Table 3), revealing the following.

**Table 3:** Quantitative CMV RT-PCR showing CMV viraemia CMV: cytomegalovirus; RT-PCR: reverse transcription polymerase chain reaction

Test name	Result
CMV quantitative detection by RT-PCR	9200 IU/ml

A diagnosis of CMV syndrome was considered, and valganciclovir 900 mg twice daily was commenced. Immunosuppression was adjusted by withholding mycophenolate mofetil and increasing the prednisolone dose.

**Figure 1:**
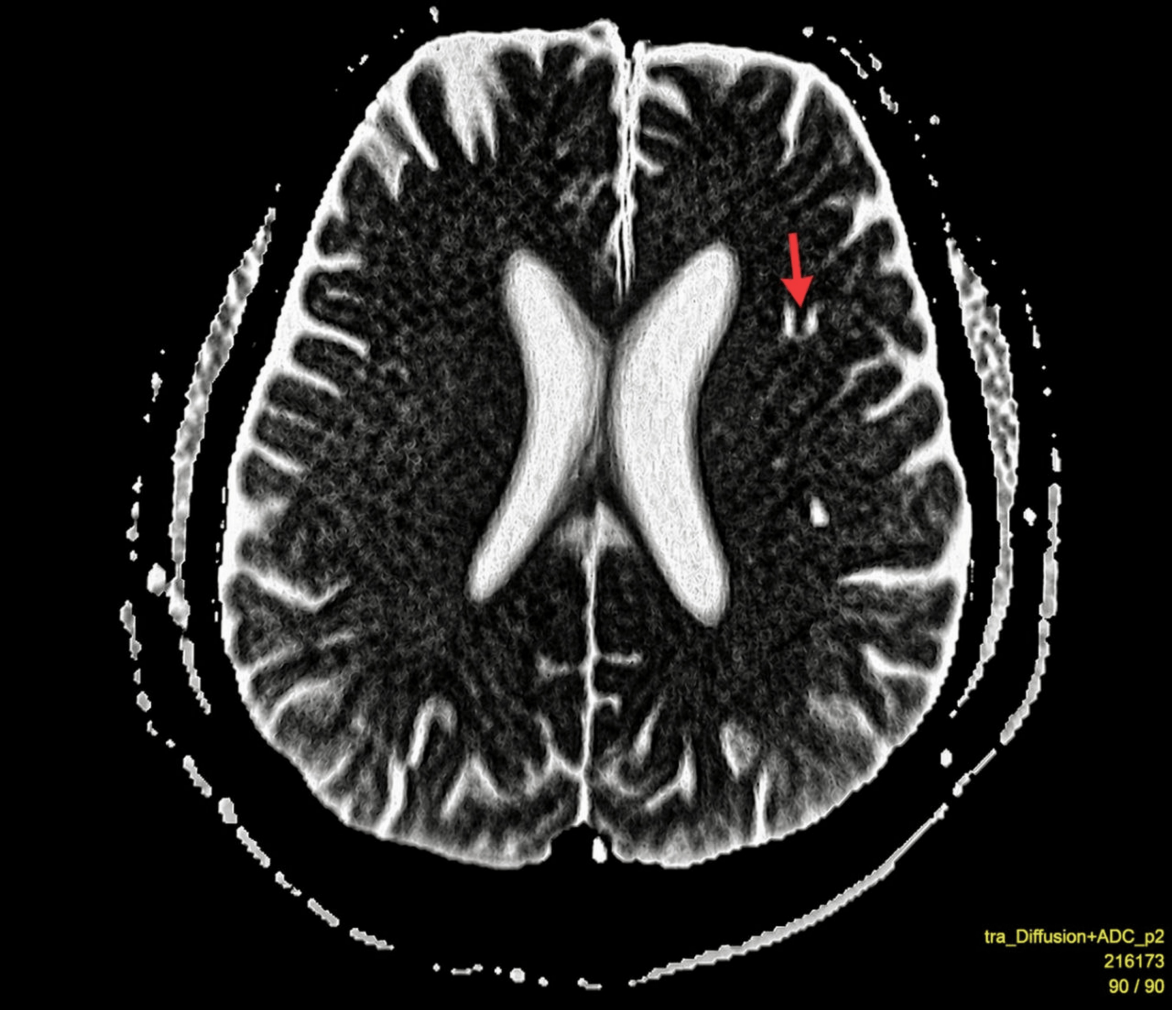
MRI of the brain An acute lacunar infarct is noted in the left external capsule. Subtle diffusion/FLAIR mismatch is present. MRI: magnetic resonance imaging; FLAIR: fluid-attenuated inversion recovery

Two days later, he developed multiple episodes of generalized seizures with worsening headache and new-onset fever. An urgent magnetic resonance imaging (MRI) of the brain was subsequently performed (Figure 1).

His MRI of the brain revealed an acute lacunar infarct in the left external capsule with subtle diffusion restriction. Additionally, lumbar puncture was performed, and the cerebrospinal fluid (CSF) was sent for biochemical analysis, cytology, meningitis/encephalitis PCR panel testing, and culture.

CSF analysis (Table 4) revealed mildly elevated protein with minimal pleocytosis and normal glucose levels, findings that were non-specific and not suggestive of bacterial meningitis. In view of the CSF result, antiviral therapy was continued.

**Table 4:** CSF analysis revealing mildly elevated protein with minimal pleocytosis CSF: cerebrospinal fluid analysis; RBC: red blood cell; WBC: white blood cell

Test name	Result	Reference range
Opening pressure	15 cmH2O	5-20 cmH2O
Glucose	3.4 mmol/L	2.22-4.44 mmol/L
Protein	0.84 g/L	0.15-0.45 g/L
Chloride	125 mmol/L	120-130 mmol/L
Total count of RBC	0	-
Total count of WBC	1	-
Polymorphonuclear cell	59%	-
Mononuclear cell	41%	-

Later on, CSF meningitis/encephalitis panel PCR (Table 5) detected *Cryptococcus neoformans*.

**Table 5:** Meningitis/encephalitis panel PCR showing the detection of Cryptococcus neoformans in the cerebrospinal fluid

Meningitis/encephalitis panel PCR		
Fungi	Cryptococcus neoformans/gattii	Detected
Viruses	Adenovirus	Not detected
	Cytomegalovirus (CMV)	Not detected
	Epstein-Barr virus (EBV)	Not detected
	Varicella-zoster virus (VZV)	Not detected
	Human parechovirus (HPeV)	Not detected
	Enterovirus	Not detected
	Herpes simplex virus type 1 (HSV 1)	Not detected
	Herpes simplex virus type 2 (HSV 2)	Not detected
	Human herpes virus 6 (HHV6)	Not detected
	Human herpes virus 7 (HHV7)	Not detected
	Mumps virus	Not detected
	Parvovirus B19	Not detected
Bacteria	*Mycobacterium tuberculosis *(MTB)	Not detected
	Mycoplasma pneumoniae	Not detected
	Streptococcus pneumoniae	Not detected
	Neisseria meningitidis	Not detected
	Haemophilus influenzae	Not detected
	Listeria monocytogenes	Not detected
	*Escherichia coli *K1	Not detected
	Streptococcus agalactiae	Not detected

The CSF culture report (Table 6) also detected *Cryptococcus neoformans*, confirming the diagnosis of cryptococcal meningitis.

**Table 6:** CSF fungal culture demonstrating the growth of Cryptococcus neoformans CSF: cerebrospinal fluid

Test name	Result
Fungal culture of CSF	Cryptococcus neoformans

A neurology opinion was sought in view of the MRI findings. In the context of active central nervous system infection and no focal neurological deficits, antiplatelet therapy was not initiated.

The patient was promptly started on liposomal amphotericin B at 3 mg/kg/day and flucytosine at 100 mg/kg/day orally in four divided doses (adjusted for renal function) for two weeks, followed by fluconazole 400 mg once daily for eight weeks. Valganciclovir was continued until CMV viraemia resolved.

Renal function and tacrolimus levels were closely monitored throughout treatment. The patient showed significant clinical improvement, becoming afebrile and seizure-free, with resolution of headache. He was subsequently discharged with a follow-up plan.

## Discussion

Cryptococcal meningitis is a serious opportunistic infection that predominantly affects immunocompromised individuals, such as those with HIV infection, malignancies, organ transplantation, autoimmune diseases, diabetes mellitus, or chronic alcoholism or those who use immunosuppressive medications [2]. In this population, clinical presentation is often subacute and non-specific. Common clinical features include neurological symptoms such as headache, changes in mental status, seizure, and neck stiffness, along with systemic signs like fever, nausea, vomiting, and lethargy [3]. Early recognition is essential, as delayed therapy is associated with increased morbidity and mortality.

Cryptococcosis is primarily caused by two species of *Cryptococcus*, namely, *Cryptococcus neoformans* (*C. neoformans*) and *Cryptococcus gattii *(*C. gattii*), with risk factors and underlying causes varying depending on the specific species involved. *C. neoformans *is widely distributed in the environment globally, while *C. gattii *is predominantly found in tropical and subtropical regions but has also been identified in cooler climates [4].

Concurrent opportunistic infections in transplant recipients have been reported but remain relatively uncommon. CMV infection, in particular, can modulate immune function and may predispose patients to additional opportunistic infections, including invasive fungal diseases [5]. In this case, significant CMV PCR positivity initially contributed to a diagnostic dilemma and may have delayed the consideration of an alternative central nervous system opportunistic infection. Cryptococcal meningitis is associated with a significant risk of lacunar stroke, especially in the basal ganglia, often leading to neurological disability among survivors [6]. These strokes are typically linked to inflammation-induced vasculitis, thrombosis, or arterial narrowing caused by the infection [7].

Management of cryptococcal meningitis requires prompt initiation of antifungal therapy. Current guidelines recommend initiating treatment for cryptococcal meningitis in HIV-infected individuals with amphotericin B, either alone or in combination with flucytosine. This should be followed by maintenance therapy with fluconazole to prevent relapse and ensure long-term management [8]. In transplant recipients, careful adjustment of immunosuppressive therapy is also essential to balance infection control with the risk of graft rejection. The patient's rapid clinical recovery following antifungal therapy highlights the importance of timely intervention even in cases with atypical presentations.

## Conclusions

This case highlights the importance of considering cryptococcal meningitis in renal transplant recipients presenting with neurological symptoms, even when initial findings suggest alternative diagnoses such as CMV infection. Clinicians should maintain a high index of suspicion for atypical and coexisting opportunistic infections to ensure timely diagnosis and appropriate management.
